# A long-acting GH receptor antagonist through fusion to GH binding protein

**DOI:** 10.1038/srep35072

**Published:** 2016-10-12

**Authors:** Ian R. Wilkinson, Sarbendra L. Pradhananga, Rowena Speak, Peter J. Artymiuk, Jon R. Sayers, Richard J. Ross

**Affiliations:** 1 The University of Sheffield, Sheffield, UK

## Abstract

Acromegaly is a human disease of growth hormone (GH) excess with considerable morbidity and increased mortality. Somatostatin analogues are first line medical treatment but the disease remains uncontrolled in up to 40% of patients. GH receptor (GHR) antagonist therapy is more effective but requires frequent high-dose injections. We have developed an alternative technology for generating a long acting potent GHR antagonist through translational fusion of a mutated GH linked to GH binding protein and tested three candidate molecules. All molecules had the amino acid change (G120R), creating a competitive GHR antagonist and we tested the hypothesis that an amino acid change in the GH binding domain (W104A) would increase biological activity. All were antagonists in bioassays. In rats all antagonists had terminal half-lives >20 hours. After subcutaneous administration in rabbits one variant displayed a terminal half-life of 40.5 hours. A single subcutaneous injection of the same variant in rabbits resulted in a 14% fall in IGF-I over 7 days. In conclusion: we provide proof of concept that a fusion of GHR antagonist to its binding protein generates a long acting GHR antagonist and we confirmed that introducing the W104A amino acid change in the GH binding domain enhances antagonist activity.

Acromegaly is a disease of growth hormone (GH) excess^1^. Patients have increased morbidity and mortality resulting from disfigurement, hypertension, type 2 diabetes, and cardiomyopathy. Primary treatment is through pituitary surgery with removal of the GH secreting tumour, but cure is only achieved in 50% of patients and those who are not cured or in whom surgery is contraindicated require medical therapy. Drug treatment of the disease, with normalisation of GH levels, returns patients to a normal life expectancy. Somatostatin analogues are the first line medical therapy for acromegaly except in very mild cases where dopamine agonist therapy may control the disease. Somatostatin acts through a family of G-coupled receptor proteins expressed on the pituitary tumour^2^. However, not all tumours express somatostatin receptors. Somatostatin-analogue therapy fails to control disease in up to 40% of patients^3^. Pasireotide, a recently developed somatostatin analogue with greater activity at certain receptor subtypes, was shown to be effective in treatment of some acromegalic patients resistant to Octreotide^4^.

The GH receptor is a cell surface transmembrane protein and a member of the type 1 cytokine receptor family. Cleavage of the extra cellular domain of its cognate receptor generates a circulating GH binding protein (GHBP). GH signalling requires that GH engages both receptor molecules that exist on the cell surface in a preformed but inactive GHR dimer. Binding of GH to its receptor triggers a conformational change resulting in signalling and internalisation (Fig. 1a), leading to the production of IGF-I. GH has two receptor binding facets: site 1 is a high affinity site while site 2 is weaker. The two binding sites on GH engage a similar region on the receptor binding protein centred around tryptophan 104 (W104) (Fig. 1b).

The discovery in 1990 that a mutation in receptor binding site 2 of GH created a GHR antagonist brought a new medical therapeutic approach to the treatment of acromegaly^5^. The pegylated GHR antagonist, Somavert (Pegvisomant), is highly effective, and controls acromegaly in >90% of patients^6^,^7^,^8^, and may improve quality of life for acromegalic patients^9^. However, Somavert either requires daily high dose injections or if combined with somatostatin analogues weekly high dose injections and is not prescribed in many countries as it is not considered cost-effective^10^. There is therefore an unmet need for the development of a long acting GHR antagonist which is cost effective with the potential for a once weekly injection regimen.

In addition to the Glyine-120 to lysine antagonistic substitution in site two, Pegvisomant has eight further amino acid changes in site 1. These result in a GH derivative that forms a high-affinity complex with one GHR molecule. Pegvisomant acts by binding to the receptor dimer and blocking signalling but also triggers internalisation^11^,^12^. Pegvisomant has been covalently modified with polyethylene glycol to increase its otherwise short *in vivo* half-life^13^. Pegylation is a chemical modification step which adds complexity to the manufacturing process and reduces affinity for GHR^11^.

We propose in this manuscript an alternative approach to delay clearance of a GHR antagonist which should be cheaper to manufacture and could generate a more cost-effective long acting GHR antagonist. We previously demonstrated that a chimeric molecule comprised of GH linked to GHBP is a potent and long-acting GHR agonist^14^. This ligand-receptor fusion had interesting characteristics. It existed in solution as both a monomer and dimer where the GH moiety of one molecule bound to the receptor portion of another molecule in a head-to-tail reciprocal dimer (Fig. 1c). We hypothesised that this was an attractive characteristic for an agonist whereby the fusion molecule provided an inactive intravascular store of GH through a dynamic equilibrium generating free agonist to act on the receptor. The pharmacokinetic characteristics suggested this fusion molecule could provide GH agonist therapy requiring injection every 21–28 days compared to daily GH administration. Thus, this technology clearly demonstrated that fusion to GH binding protein is an effective technology to delay clearance^15^. We have now tested whether a fusion of the GHR antagonist molecule to the GH binding protein could generate a long acting GHR antagonist and the hypothesis that introducing an amino acid change in the GH binding domain (W104A) would increase bioactivity. We produced 3 molecules, GHA1-3 (Fig. 1d), and tested them *in vitro* and *in vivo*. The results demonstrate that this approach generates a GH antagonist with delayed clearance and biological activity *in vivo*.

## Results

### Protein expression, purification and stability

GHA1-3 were expressed in CHO cells grown in roller bottle cultures in the presence of 2 mM valproic acid at a set temperature of 31 °C and purified by antibody affinity chromatography. All three proteins were >95% pure as judged by SDS-PAGE. Stability studies conducted on all proteins showed no degradation over 8 days at 4 °C, room temperature and −80 °C with multiple freeze thaw cycles.

### *In vitro* bioactivity

*In vitro* bioactivity was tested using a dual luciferase transcription assay based on the induction of firefly luciferase expression by GH. Transfected cells were incubated in a constant concentration of GH and with an increasing concentration of GHR antagonist. GHA3 showed the greatest inhibition of GH activity and the order of greatest inhibition to least was GHA3 (mean ± sem: IC_50_ = 17.1 ± 1.3 nM) > GHA1 (IC_50_ = 44.4 ± 4.7 nM > GHA2 (IC_50_ = 198.4 ± 25.2 nM). A 1-way ANOVA showed significance difference between treatments, p < 0.0001. Carrying out a post-hoc parametric unpaired 2-tailed t-test showed that there were significant differences between treatments with p values = 0.0002 for GHA1 vs. GHA3 and p values < 0.0001 for GHA2 vs. GHA1 and GHA3 respectively. The alpha value was set at 0.05.

### Pharmacokinetics

**Figure 2:**
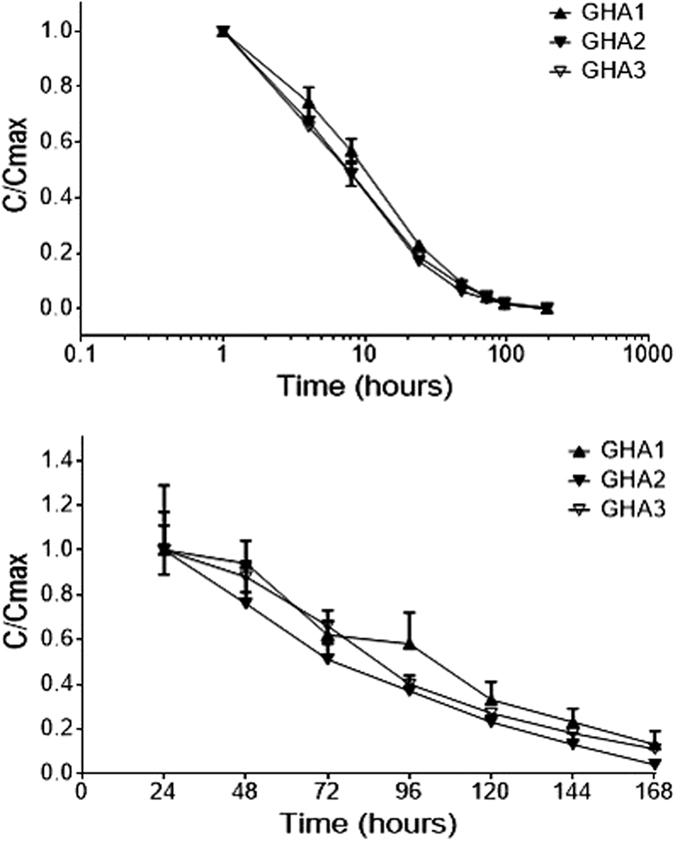
Pharmacokinetic studies in rats and rabbits. **(a)** Pharmacokinetics in rats. A single bolus intravenous injection of 1 nmole/kg was given to Crl:CD(SD) male rats (n = 6 animals per group). Sera was taken at specified time points and assayed for the presence of GHA. Data is displayed as a ratio of C/C_max_ (+/− SEM). **(b)** Pharmacokinetics in rabbits. A single subcutaneous injection of 2 mg/kg was given to New Zealand White male rabbits (n = 3 animals per group). Sera was taken at specified time points and assayed for the presence of GHA. Data is displayed as a ratio of C/C_max_ (+/− SEM).

After i.v. administration in rats GHA1, 2 and 3 had similar terminal half-lives of >20 hours (Fig. 2a and Table 1a). After s.c. administration in rabbits all molecules had a T_max_ at 24 hours and GHA3 had a terminal half-life of 40.5 hours (Fig. 2b and Table 1b).

### *In vivo* bioactivity

**Figure 3:**
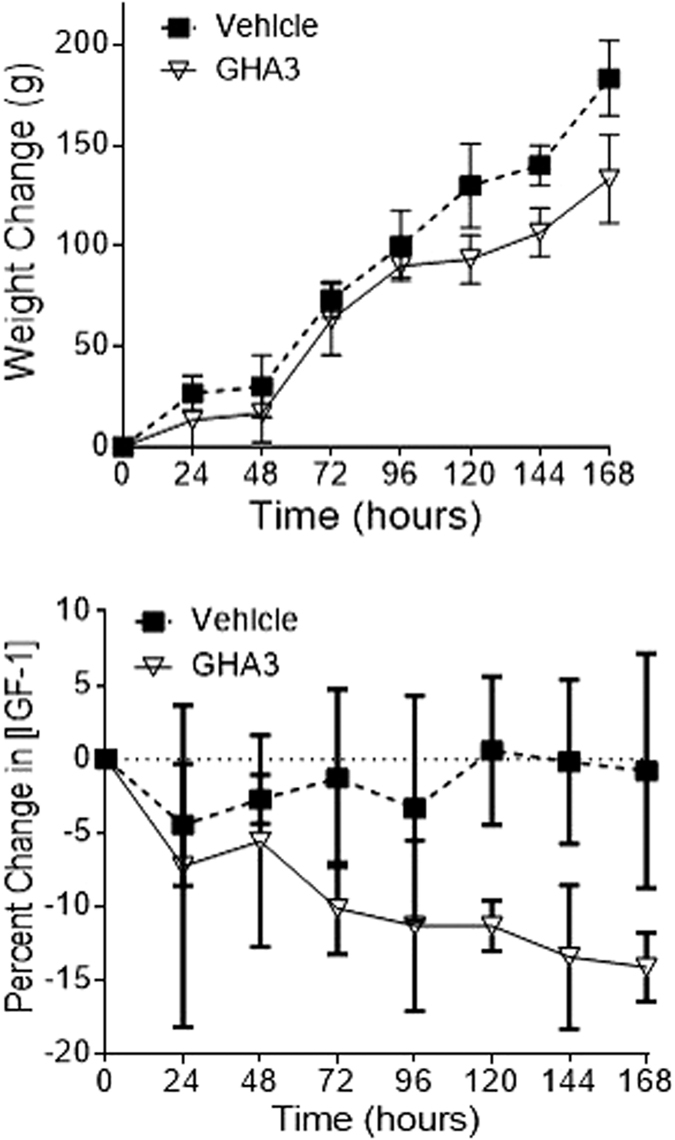
Pharmacodynamics of GHA3 in New Zealand White Male Rabbits. A single subcutaneous injection of 2 mg/kg was given to New Zealand White male rabbits (n = 3 animals per group). A buffer only vehicle control was also tested (n = 3 animals per group).The pharmacodynamic effects of GHA3 were followed over 196 hours (7 days). **(a)** Concentrations of IGF-I from rabbit serum samples challenged with GHA3 (+/− SEM). Samples were analysed using an automated iDS human IGF-I ELISA. **(b)** Body weight measurements were obtained daily over the study period.

Body weight and IGF-I were measured in rabbits after a single injection of GHA3 compared to vehicle only with n = 3 animals per group. GHA3 induced a fall in IGF-I by 14% after s.c injection in rabbits at 7 days (Fig. 3a) which was associated with a loss in body weight gain (Fig. 3b).

## Discussion

We have demonstrated that a GHR antagonist generated through an amino acid change in the site 2 binding site of GH for its receptor has delayed clearance when fused to GH binding protein. It is absorbed after subcutaneous injection and reduces IGF-I levels in the rabbit after a single injection. This provides proof of concept for a second generation long acting GHR antagonist. An advantage of this technology is no requirement for chemical modification after purification such as pegylation.

We tested 3 different GH molecules, GHA1-3, all of which had a G120R amino acid change in receptor binding site 2 of the GH molecule that generates a competitive GHR antagonist. Pegvisomant has 8 amino acid changes in binding site 1 to enhance its binding to the receptor^13^, and we therefore introduced these changes in to our molecule GHA2, however this resulted in a reduction in antagonist activity. We predicted this might be the case as enhancing binding of site 1 to the receptor would likely also increase both an intra- and intermolecular binding of the GH moiety in our fusion molecule to the GH binding protein moiety. To prevent intra- and inter-molecular binding of our fusion molecule we introduced an amino acid change in a critical residue, W104, in the GH binding protein moiety. The introduction of W104A amino acid change in association with the site 1 and 2 amino acid changes in molecule GHA3 greatly increased GHR antagonist activity. This was demonstrated in the bioassay where GHA3 showed the greatest inhibition of GH activity and the order of greatest inhibition to least was GHA3 > GHA1 > GHA2.

The terminal half-life of all our fusion molecules was greatly prolonged when compared to GH which has a terminal half-life of 1.4 h in rats^16^ In the rat all our GHA molecules had terminal half-lives >20 hours. Similar results were seen after subcutaneous injection in the rabbit with GHA3 having the most delayed clearance although it should be noted that the results are for the terminal half-life and that in the rabbits the first sample taken was at 24 hours and it is possible the Tmax preceded this time-point. These results were very similar to those we had previously found for a GHR agonist fusion with GH binding protein^14^. We had previously considered that the delayed clearance of our GHR agonist fusions may relate to their ability to form head to tail reciprocal dimers in solution; however the demonstration that introducing the W104A amino acid change did not affect clearance suggests that the monomeric molecule has delayed clearance. It appears that adding certain proteins to the C-terminus of GH such as albumen, CTP, Fc fragment and XTEN can delay clearance^17^. We previously undertook pharmacokinetic studies with our GHR agonist in non-human primates and based on allosteric modelling calculated that the fusion would only need to be administered once every 3 weeks^15^. Accepting that a GHR antagonist is required at higher concentration we can anticipate that GHA3 may only need to be administered once a week.

One of the challenges in developing human GHR antagonists has been identifying an appropriate animal model for testing. The original observation that a site 2 amino acid change in GH created a GHR antagonist was made in a transgenic mouse model^5^, however the exogenous administration of human GH or human GHR antagonist to rodents has little effect on IGF-I levels^18^. To address this we have investigated using rabbits as a model for GH bioactivity and demonstrated that GH increased and Pegvisomant decreased IGF-I levels in the rabbit^19^. We therefore tested GHA3 in rabbits and it caused a 14% fall in IGF-I levels 7 days after a single subcutaneous injection and this was associated with a reduction in weight gain.

In conclusion we have tested three different GHR antagonist molecules and provide proof of concept that a fusion of a GHR antagonist with amino acid changes in site 1 to enhance binding and amino acid changes in the GH binding protein moiety to reduce intra and inter-molecular binding provides a potent GHR antagonist with the potential for weekly dosing.

## Methods

### Construction of GHR Antagonists

Three molecules were constructed by a combination of gene synthesis (GeneCust Ltd, France) and standard DNA manipulation techniques. Recombinant genes encoding full length GH antagonist were cloned into a modified mammalian expression plasmid, pSecTag/FRT/V5/Hist-TOPO (Invitrogen). Stable cell lines were produced in the CHO Flp-In cell line (Invitrogen) according to manufacturer’s instructions and adapted to serum free media in Hyclone SFM4CHO Utility (Thermo Scientific).

### Expression and Purification

Cells were maintained in roller bottle cultures in Hyclone SFM4CHO Utility medium with passaging every 2–3 days, keeping cell densities between 0.25 × 10^6^ viable cells/ml (VCPM) and 1.5 × 10^6^ VCPM. For expression studies, roller bottles were seeded at 0.5 × 10^6^ VCPM and grown at 37 °C, 5% CO_2_ and allowed to reach 1 × 10^6^ VPCM. Valproic acid was added to a final concentration of 2 mM and the temperature reduced to 31 °C. Cells were grown until viability was ~30–40% then clarified by centrifugation at 22,000 x *g* using a Beckman JLA 16–25 rotor for 20 minutes at 4 °C. EDTA and Benzamidine-HCl were added to final concentrations of 5 and 10 mM respectively and the medium concentrated using a Vivaflow 200 tangential flow concentrator and stored frozen at −20 °C. Target protein was purified from this concentrate by affinity chromatography. Briefly an anti-GH antibody (5E1, a kind gift from Prof. Christian Strasburger, Berlin, Germany) was coupled to 80 ml of NHS-activated Sepharose (GE Healthcare), packed into an XK 16/50 chromatography column (GE Healthcare) and equilibrated in phosphate-buffered saline (PBS). Frozen supernatant was rapidly defrosted at 37 °C and centrifuged at 25,000 x *g* for 30 minutes at 4 °C using a Beckman JA 25–50 rotor. A 1/10 volume of 10x concentrated PBS was added and sample loaded onto the column at 5 ml/min at 4 °C. Post-binding the column was washed with 10 column volumes (CV) of PBS. Bound protein was eluted using 0.2 M glycine, pH 2.7. The elution fractions were immediately neutralised with 1/10 volume of 1.5 M Tris, pH 8.8. Samples were analysed by SDS-PAGE. Fractions containing purified protein were dialysed against Citrate buffer (20 mM sodium citrate, 10% (v/v) glycerol, 0.15 M NaCl, pH 6.5) followed by concentration using a Centriprep-30 concentrator (Millipore Inc.). Protein concentrations were measured by Bradford protein assay and samples aliquoted and stored at −80 °C.

### Stability Studies

For stability studies purified samples were first filtered (0.22 μm filter) followed by dilution to 1 mg/ml with 20 mM Citrate buffer, pH 6.5, 0.15 M NaCl, 10% glycerol. Samples were incubated at room temperature, 4 °C and −80 °C for a total of 8 days with samples taken on days 0, 1, 4 and 8. All samples were analysed by SDS-PAGE under non-reducing conditions followed by visualization with Coomassie blue staining.

### GH Sandwich ELISA

GHR antagonist levels were routinely quantified using an in-house ELISA developed to detect GH and GHA1-3. The method is based on a sandwich ELISA format utilising a coating antibody (Monoclonal antibody 7F8) and a biotinylated secondary antibody (Monoclonal antibody 10A7). Both antibodies were a kind gift from Prof. Christian Strasburger, Berlin, Germany. 10A7 was biotinylated in-house using No-weigh NHS-Biotin (Thermo Scientific) at a molar ratio of 400:1 (biotin: antibody). Detection was by Streptavidin-HRP (GE Healthcare) using Superslow Kinetic TMB (Sigma, UK). The reaction was stopped by the addition of 5% (v/v) sulphuric acid and the plates read at 450 nm with correction at 630 nm. For detection of GHA from *in vivo* serum samples, the assay was conducted in the presence of 5% (v/v) rat or rabbit serum (Sigma, UK) to account for matrix effects. Unless otherwise stated standard curves were derived using the relevant specific purified GHA1-3 molecules.

### Dual Luciferase Bioactivity Assay

The dual luciferase assay was used to assess the GHR antagonist’s ability to inhibit the actions of GH and has been described previously. Briefly, HEK293 cells stably expressing the full length growth hormone receptor were routinely grown in DMEM:F12 (Gibco) supplemented with 10% FCS (Thermo Scientific), 2 mM Glutamine (Gibco), 1% penicillin/streptomycin (Gibco) and 250 μg/ml Hygromycin B (Invitrogen). For the assay, cells were plated at 2 × 10^5^ VCPM in a 24 well cell culture dish in the above media without Hygromycin B, and allowed to adhere overnight at 37 °C, 5% CO_2._ The next day cells were transfected with two reporter plasmids, one containing firefly luciferase, whose expression is under the control of a STAT5 response element linked to a thymidine kinase minimal early promoter and the other a transfection control plasmid expressing *Renilla* luciferase, under the constitutive control of a CMV promoter. For the assessment of GHR antagonist activity, transfected cells were incubated with 0.25 nM GH together with increasing concentrations of GHA1-3 for 6 hours at 37 °C, 5% CO_2_. Both firefly and *Renilla* luciferase levels were measured using a dual Luciferase Assay System (Promega) and analyzed using an Autolumat Plus Luminometer (Berthold Technologies).

### *In vivo* properties of GHA1-3

All animal studies were conducted by Sequani Limited in accordance with all applicable sections of the United Kingdom Animals Act 1986 and the associated Codes of Practice for the Housing and Care of Animals used in Scientific Procedures and the Humane Killing of Animals under Schedule 1 to the Act, issued under section 21 of the Act. All experimental protocols were approved by Sequani Limited through a local ethical review process, as required under the Animals (Scientific Procedures) Act 1986. This process aims to ensure that animal use is carefully considered and fully justified, proper account is taken of all possibilities for reduction, refinement and replacement and the highest practicable standards of care, health and ethics are maintained in animal based research.

### Pharmacokinetic profiles of GHA1-3 in Rats

Male Sprague Dawley rats (n = 6 per group) were injected once by intravenous (bolus) injection with 1 nMole/kg of GHA1-3 in 20 mM Citrate buffer, pH 6.5, 0.15 M NaCl, 10% glycerol or vehicle control only which was 20 mM Citrate buffer, pH 6.5, 0.15 M NaCl, 10% glycerol. Sampling was at time zero (Pre-dose) and at 1, 4, 8, 24, 48, 72, 96 and 192 hours post-dose. Serum samples were analysed using an in-house GH ELISA in the presence of 5% rat serum. Pharmacokinetic parameters were derived from the individual serum concentration-time profiles by non-compartmental analysis of the individual data using a validated system. Nominal sampling times were used for all calculations of pharmacokinetic parameters. Calculations were performed by the linear trapezoidal method, using raw data. Pharmacokinetic parameters are displayed to three significant digits, except t_½_ which is expressed to one decimal place, and T_max_ which is displayed as nominal time. Group mean values were calculated for each group separately, as appropriate.

### Pharmacodynamic profiles of GHA3 in Rabbits

Male New Zealand white rabbits (n = 3 per group) were injected with a single subcutaneous injection of 2 mg/kg GHA in 20 mM Citrate buffer, pH 6.5, 0.15 M NaCl, 10% glycerol or vehicle control only which was 20 mM Citrate buffer, pH 6.5, 0.15 M NaCl, 10% glycerol. Sampling was at time zero (Pre-dose) and at 24, 48, 72, 96, 120, 144 and 168 hours post-dose. Serum samples were analysed using an in-house GH ELISA in the presence of 5% rabbit serum. IGF-1 levels were analysed using the iDS SYS human IGF-1 automated assay that had previously been validated for the detection of Rabbit IGF-1^19^. Pharmacokinetic parameters were derived from the individual serum concentration-time profiles by non-compartmental analysis of the individual data using a validated system. Nominal sampling times were used for all calculations of pharmacokinetic parameters. Calculations were performed by the linear trapezoidal method, using raw data. Pharmacokinetic parameters were displayed to three significant digits, except t_½_ which was expressed to one decimal place, and T_max_ which was displayed as nominal time. Group mean values were calculated for each group separately, as appropriate.

### Statistical analysis

Comparison of bioactivity between the GHR antagonists was made by ANOVA with post-hoc analysis by a parametric unpaired 2-tailed t-test. The alpha value was set at 0.05.

## Additional Information

**How to cite this article**: Wilkinson, I. R. *et al*. A long-acting GH receptor antagonist through fusion to GH binding protein. *Sci. Rep.* **6**, 35072; doi: 10.1038/srep35072 (2016).

## Figures and Tables

**Figure 1:**
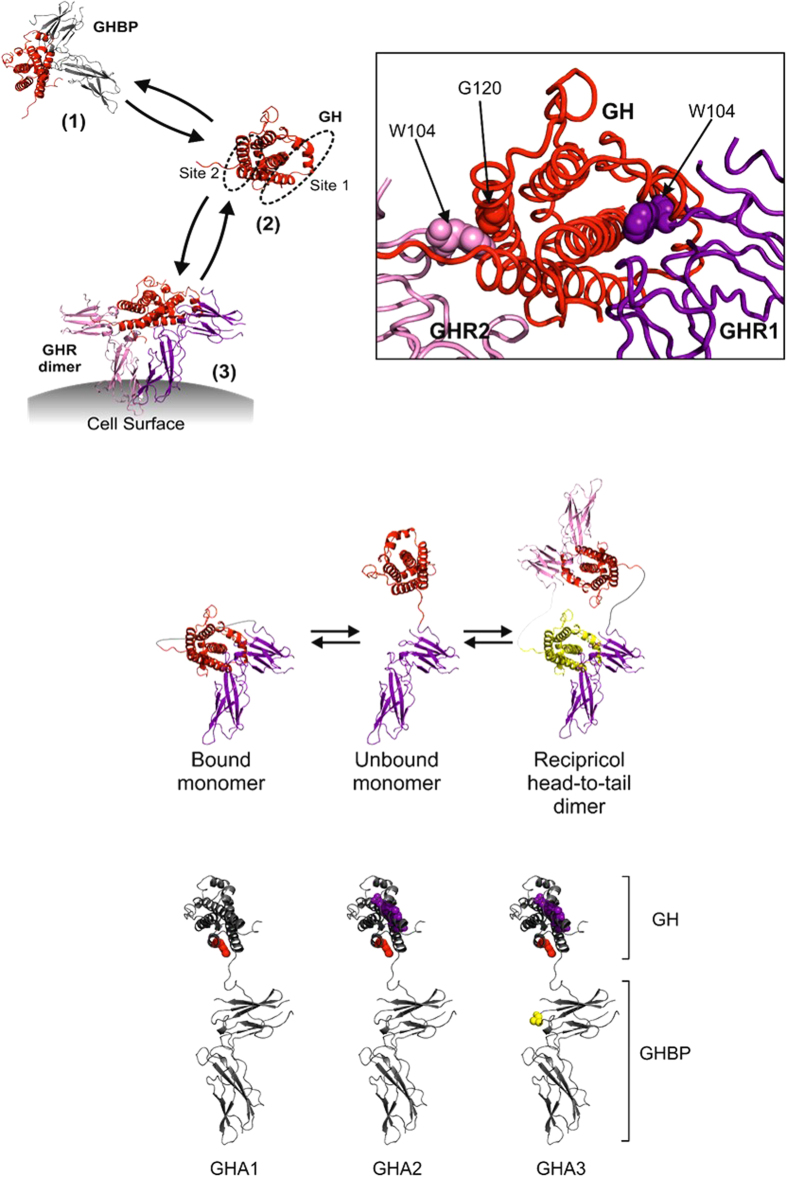
Overview of molecular interactions of GH and GH-Fusions. (**a**) Schematic showing the binding of GH to growth hormone binding protein (GHBP) (1).GH binding is via two binding sites: site 1 (high affinity) and site 2 (low affinity) (2). GH binds to preformed GH receptor (GHR) dimers at the cell surface (3). (**b**) Detailed view of the GH/GHR interfaces, GH (red) is bound to two GHR molecules at site 1 (GHR1) and site 2 (GHR2). A G120R amino acid change (red spheres) abolishes binding at site 2; and the GHR W104A amino acid change (pink and purple spheres) abolishes binding to GH. (**c**) Possible conformations of the GH ligand-receptor fusions. The GH domain could associate via intramolecular interactions with GHBP to form an inactive closed monomer conformation, exist as an open monomeric conformation or it could form a reciprocal head-to-tail dimer. (**d**) GHA1-3 antagonist molecules consisting of GHR antagonist linked directly to the N-terminus of GHBP. GHA molecules contain combinations of GH site 1 amino acid changes - H18D, H21N, R167N, K168A, D171S, K172R, E174S and I179T (purple), GH site 2 (G120R) amino acid change (red), and the GHR W104A amino acid change (yellow).

**Table 1:** Pharmacokinetics of GHA1-3 Rats and Rabbits.

a			
	*GHA1*	*GHA2*	*GHA3*
*C*_*max*_ (*ng*/*mL*)	407	699	1020
*C*_*0*_ (*ng*/*mL*)	453	797	1180
***T***_***1*****/*****2***_ (***h***)	**20.4**	**20.6**	**21.2**
**b**			
*C*_*max*_ (*ng*/*mL*)	6740	7190	5410
*T*_*max*_ (*h*)	24	24	24
***T***_***1/2***_ (***h***)	**23.6**	**17.4**	**40.5**

(**a**) A single bolus intravenous injection of 1 nMole/kg was given to male rats (n = 6 animals per group). (**b**) A single subcutaneous injection of 2 mg/kg was given to New Zealand White male rabbits (n = 3 animals per group).
